# Genome sequence of the aurodox-producing bacterium *Streptomyces goldiniensis* ATCC 21386

**DOI:** 10.1099/acmi.0.000358

**Published:** 2022-08-19

**Authors:** Rebecca E. McHugh, John T. Munnoch, Andrew J. Roe, Paul A. Hoskisson

**Affiliations:** ^1^​ Strathclyde Institute of Pharmacy and Biomedical Science, University of Strathclyde, 161 Cathedral Street, Glasgow, G4 0RE, UK; ^2^​ *Institute of Infection, Immunity and Inflammation, University of Glasgow, Glasgow, G12 8TA, UK

**Keywords:** Streptomyces, Aurodox, elfamycin, antimicrobial, genome

## Abstract

We report the genome sequence of *Streptomyces goldiniensis* ATCC 21386, a strain which produces the anti-bacterial and anti-virulence polyketide, aurodox. The genome of *S. goldiniensis* ATCC 21386 was sequenced using a multiplatform hybrid approach, revealing a linear genome of ~10 Mbp with a G+C content of 71%. The genome sequence revealed 36 putative biosynthetic gene clusters (BGCs), including a large region of 271 Kbp that was rich in biosynthetic capability. The genome sequence is deposited in DDBJ/EMBL/GenBank with the accession number PRJNA602141.

## Introduction

Isolated from soil collected in Bermuda in 1973, a novel strain of *Streptomyces* was found to produce the anti-Streptococcal natural product (X-5108=aurodox) and was informally named *S. goldiniensis var*. *goldiniensis* [1]. Despite the strain being named *S. goldiniensis var*. *goldiniensis* by Berger *et al*. [1], there is no formal description of this strain in the literature and it does not appear in the List of Prokaryotic names with standing in nomenclature [2].

Originally identified for its antibacterial activity, aurodox [1] has also found utility as a widely used growth promoting compound in poultry [3]. More recently it has attracted attention for its anti-virulence activity, blocking virulence by inhibition of the Type III Secretion System (T3SS) in Enterohaemorrhagic *Escherichia coli* (EHEC) [4]. Subsequent work this inhibitory effect is mediated through the down-regulation of the Type III Secretion System master regulator Ler [5].

Whilst *S. goldiniensis var*. *goldiniensis* is known for the production of aurodox, it is a common feature of *Streptomyces* genomes to encode many more secondary metabolites than can be observed during laboratory culture [6]. The vast repository of natural product biosynthetic gene clusters (BGCs) contained within the genomes of *Streptomyces* species means that genome mining has become the mainstay of researchers looking to prioritize BGCs for further study [6, 7]. Moreover, the increasing amount of genome sequence data of natural product producing strains facilitates evolutionary studies of biosynthesis and can inform on synthetic biology strategies for developing novel molecules and improving production of current molecules [6–8]. Here we describe the multi-platform genome sequencing of *Streptomyces goldiniensis* ATCC 21386 which is deposited in DDBJ/EMBL/GenBank with the accession number PRJNA602141.

## Methods

### Whole genome sequencing *Streptomyces goldiniensis*.

Genomic DNA was extracted according to Kieser *et al.,* [9] from cultures grown in GYM medium (DSMZ medium 65; www.dsmz.de). Nanopore sequencing was performed using the Nanopore 1D ligation protocol with MinION SPOT ON MK1 R9 flow cells. Raw data was converted using MinKnow base calling software. Illumina platform data was provided by Microbes NG (Birmingham, UK) from the HiSeq 2500 sequencing platform. Reads were trimmed using Trimmomatic 0.30 [10] with quality cut off of Q15. PacBio sequencing was provided by (Nu-omics, University of Northumbria, UK) using the Sequel instrument with contigs assembled in HGAP4. Raw read data (Illumina, PacBio and ONT) is deposited in the Sequence Read Archive (SRA) at https://dataview.ncbi.nlm.nih.gov/object/SRR18547533, https://dataview.ncbi.nlm.nih.gov/object/SRR18547532 and Figshare https://doi.org/10.6084/m9.figshare.19487693.v2

The SPAdes platform was used to create a combined assembly using data from all three technologies. This allowed for the use of a k-mer dependent approach whilst using the initial PacBio assembly as ‘trusted contigs’ [11]. AutoMLST [12] was used to identify *S. bottropensis* ATCC 25435 (Taxonomy ID: 1054862) as the closest neighbour for scaffold-based assembly using MeDuSa [13] and quality analysis performed using QUAST [14]. Prokka was used to annotate the genome of *Streptomyces goldiniensis* [15] and is available on Genbank (Bioproject PRJNA602141). Identification of biosynthetic gene clusters was performed using the antiSMASH pipeline (bacterial version 5.0.0) [16]. The position of the putative aurodox BGC within the larger 273 Kbp ‘supercluster’ was confirmed via PCR. The following oligonucleotide primer sequences were used; clusterposcheckAF 5′-CCAGACGCAGGTCCGCTTCGGACG-3′; clusterposcheckAR 5′-CCATCGTGGGGATCGCAG-3′; clusterposcheckBF 5′- AGGATGTTCCAGTCGGCTCTCACTCCG-3′; clusterposcheckBR 5′-CGAGGTCGCCCGGCATGTGGA-3′.

PCR products were visualised using agarose gel-electrophoresis, and sequence specificity was confirmed by band excision followed by Sanger sequencing provided by Eurofins genomics (Luxembourg).

## Results and discussion

### Genome features of *Streptomyces goldiniensis* ATCC 21386

**Fig. 1.**
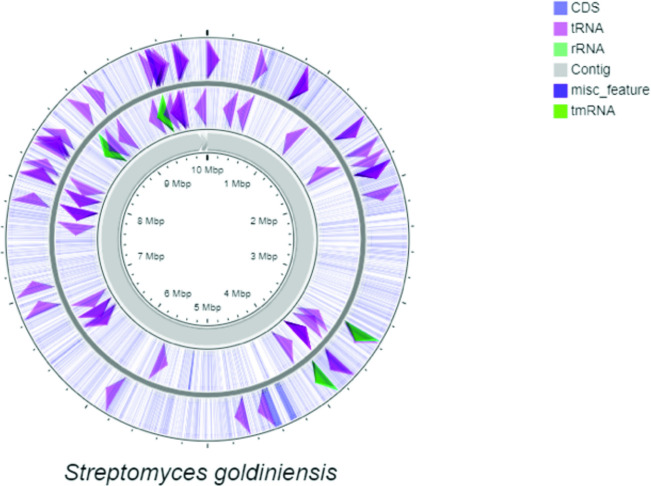
Circular representation of the linear *Streptomyces goldiniensis* genome generate by CGviewer [19]. Map was built from a .gbk file generated by Prokka. The outer most circle represents coding sequences on the major strand, the inner circle represents coding sequences on the minor strand. Grey arrows in the centre of the figure depict the lengths of the contigs in the final assembly.

The linear genome of *S. goldiniensis* ATCC 21386 was sequenced using a hybrid-approach of Illumina, PacBio and Oxford Nanopore to generate a high-quality draft genome (Genbank Bioproject PRJNA602141). Using a combined assembly approach with SPAdes [11] the data from all three platforms allowed the overall genome size to be estimated at 10005022 bp, in nine contigs, with an N50 of 9950726 bp. The draft genome of *S. goldiniensis* is predicted to have a total of 9925 protein coding genes, along with 81 tRNAs and five rRNA operons (Fig. 1). Absence of genes encoding plasmid replication machinery (Par proteins) on the eight minor contigs suggest that they do not represent plasmids. Pulse-field gel electrophoresis of total DNA extractions from *S. goldiniensis* also indicates the absence of plasmids in this strain.

### The *S. goldiniensis* genome is rich in natural product biosynthetic gene clusters

*Streptomyces* bacteria are renowned for their ability to synthesis a wide range of natural products, many of which have found utility in human medicine [7]. Despite many strains being identified through the production of a single metabolite, the genomes of *Streptomyces* often encode a number of additional biosynthetic gene clusters (BGCs) that are not expressed under laboratory conditions, the so called ‘silent BGCs’ [6]. Using the antiSMASH pipeline [16] the genome of *S. goldiniensis* ATCC 21386 was mined. A total of 36 putative BGCs were identified (Table 1), including five putative polyketide synthase (PKS) containing BGCs, eight non-ribosomal peptide synthase (NRPS) BGCs and nine putative terpene BGCs. The genome encodes BGCs that are highly conserved in the genomes of *Streptomyces* species such as geosmin, desferrioxamine and melanin [7].

**Table 1.** Predicted Biosynthetic Gene Clusters of *S. goldiniensis* using antiSMASH [17]

Region	Type	Region	Most similar	% similarity
1	Type II PKS	32,278–104 355	Spore pigment	83
3	Lanthipeptide, lassopeptide	486,431–514 192	Citrulassin E (ripp)	100
4	Siderophore	2,159,777–2 170 184	n/a	0
5	Terpene	2,487,747–2 508 882	n/a	4
6	NRPS, tfuA related	2,543,021–2 606 769	Diisonitrile antibiotic SF2768	66
7	Bacteriocin	2,705,875–2 716 826	n/a	0
8	Terpene	2,777,350–2 799 499	Geosmin	100
9	NRPS-like	2,818,277–2 858 728	S56P1	11
10	NRPS-like, siderophore	3,085,338–3 126 119	Paulomycin	13
11	NRPS-like	3,541,716–3 582 164	Octacosamicin	12
12	Terpene	3,625,827–3 643 980	n/a	0
13	Indole	4,158,688–4 181 228	Terfestatin	28
14	bacteriocin,bottromycin,NRPS,transAT-PKS, Type I PKS, hglE-KS,lassopeptide	4,213,370–4 484 508	Kirromycin/bottromycin	40
15	NRPS-like	4,599,349–4 657 342	Echosides	17
16	Terpene	4,783,720–4 802 511	n/a	0
17	Linardin	5,046,397–5 067 017	Pentostatine	17
18	Siderophore	5,085,773–5 100 346	n/a	0
19	NRPS, melanin	5,167,798–5 232 264	Scabichelin	100
20	Terpene	5,257,095–5 277 644	n/a	0
21	NRPS, Type I PKS	5,284,180–5 346 580	n/a	4
22	Terpene	5,480,336–5 505 711	Isorenieratene	100
23	Type II PKS	5,770,343–5 856 353	Fluostatins	60
24	Lanthipeptide, bacteriocin	6,153,412–6 179 099	Informatipeptin	100
25	NRPS-like, Type I PKS	6,410,852–6 457 973	Marineosin	45
26	Terpene	6,507,863–6 532 462	Hopene	100
27	Ectoine	6,852,064–6 861 735	Ectoine	100
28	Ladderane	6,937,157–6 978 500	Colabomycin	11
29	Type III PKS, Terpene	7,764,486–7 805 661	Merochlorin	12
30	Melanin	8,189,939–8 200 406	Melanin	80
31	Siderophore	8,344,177–8 355 949	Desferrioxamine A	83
32	Ectoine	8,471,171–8 481 599	Ectoine	75
33	Terpene	9,170,031–9 191 797	n/a	0
34	Bacteriocin	9,838,044–9 848 331	n/a	0
35	Terpene	9,968,421–10 005 017	n/a	0

**Fig. 2.**
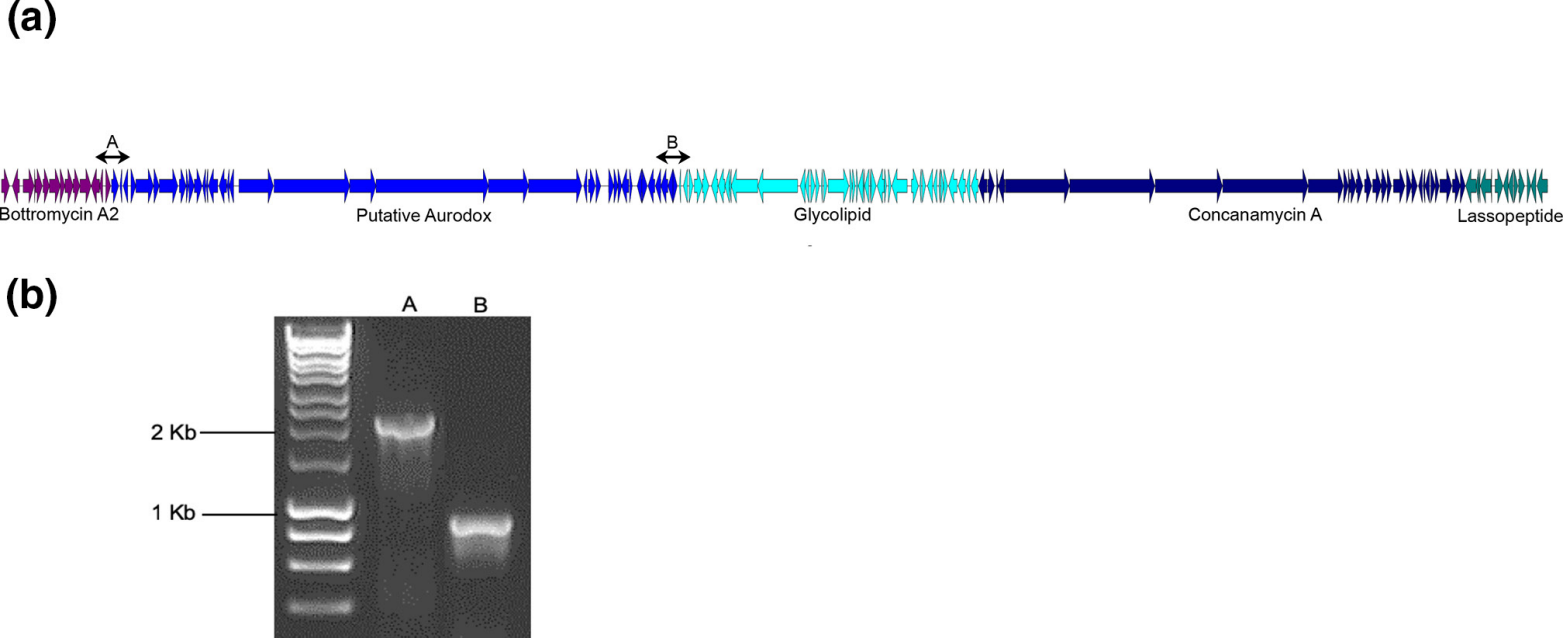
(a): Representation of the 271 kbp putative aurodox supercluster. Gene cluster boundaries were predicted by AntiSMASH and schematic diagram was generated by clinker. From left to right, arrows coloured purple represent Bottromycin A2-associated genes, blue arrows represent the putative aurodox BGC, cyan arrows represent predicted glycolipid-synthase genes, navy blue arrows represent concanamycin A-associated genes and green arrows indicate putative genes involved in lassopeptide biosynthesis. (**b):** Primer pair binding sites are indicated by ‘a’ and ‘b’ on the diagram and their corresponding PCR products are shown on the gel with the lanes labelled accordingly.

One region of the *S. goldiniensis* genome was found to be particularly rich in BGCs (position 4213370–4484508; 271 kb), encoding a putative bottromycin A2-like molecule, an 87 kb region that possesses genes likely to encode a hybrid PKS/NRPS, highly similar to the kirromycin BGC [17] which has recently been shown to encode aurodox [18]. Immediately downstream of the aurodox BGC is a gene cluster with homology to glycolipid synthase-like PKS containing BGCs, a cluster which is 100 % identical to the macrolide concanamycin A and a putative lassopeptide-encoding gene cluster (Table 1, Fig. 2). To confirm that this BGC-rich region around the aurodox BGC was indeed a supercluster, rather than an artefact of genome assembly, primer pairs were designed that spanned the junction between the cluster upstream (putative bottromycin A2) and downstream (glycolipid synthase-like PKS containing BGC) of the aurodox cluster. PCR and sequencing of the regions spanning the BGCs confirmed the organisation of the gene clusters around aurodox (Fig. 2).

## Conclusions

The genome of *S. goldiniensis* ATCC 21386 was sequenced using a hybrid approach to yield a high-quality draft genome with ~99 % of the genome on a single contig through a k-mer dependant assembly using SPAdes [11], followed by a scaffold-based final assembly with MeDuSa [13]. This allowed prediction of a biosynthetic gene supercluster to be identified from the main genome contig and the organisation of the supercluster to be confirmed by PCR. This genome sequence provides a springboard for further study of this strain and a basis for a formal taxonomic description of *S. goldiniensis var*. *goldiniensis* ATCC 21386.
